# Editorial: Physiological traits and stress detection in crops during global climate change: availability and sustainable use of water resources

**DOI:** 10.3389/fpls.2024.1371044

**Published:** 2024-02-02

**Authors:** Alfredo E. Rubio-Casal, Mohamed F. M. Ibrahim

**Affiliations:** ^1^ Department of Plant Biology and Ecology, University of Seville, Seville, Spain; ^2^ Department of Agricultural Botany, Faculty of Agriculture, Ain Shams University, Cairo, Egypt

**Keywords:** crop, drought stress, agricultural productivity, plant physiology, detecting stress

Water is the most limiting natural resource in the production of agricultural systems worldwide. The present climate change scenario predicts an increase in global temperatures, - the increasingly persistent occurrence of torrential rains, and prolonged periods of drought (IPCC, 2023). All these factors have a negative global impact on agricultural productivity. In order to adapt to these events, several actions must be carried out: 1) sustainable use of water resources to avoid the reduction of available water; 2) studies to detect drought stress in crops, and 3) research work that focuses on the consequences that moderate and extreme drought can cause in species of agronomic use.

A large number of physiological processes are affected by drought and lack of water availability. These adverse physiological impacts affect plants in different ways throughout their phenology, leading to loss of vigor, lower quantity and quality of biomass production, lower fruit number, size, and quality, and lower seed production and seed characteristics (Dietz et al., 2021).

Plants have many mechanisms to avoid the effects of lack of water availability. These include structural modifications such as cuticle development to reduce water loss, increased root growth, and variation of vascular tissue, as well as physiological adjustments such as regulation of stomatal aperture to increase water use efficiency without compromising photosynthesis. For all this, plants possess several hormone-mediated signalling mechanisms that regulate all these processes (Iqbal et al., 2022).

Ultimately, all these responses are mediated by molecular regulatory mechanisms that involve the expression of drought resistance genes generating signal transduction in which a large number of metabolites of different nature are involved; proteins such as late embryogenesis abundant, drought compatible osmolytes such as proline, betaine glycine and signalling molecules such as Ca^2+^, abscisic acid, etc… (Yang et al., 2021).

One of the current measures is the study of highly resilient varieties to global climate change. Studies focus on many dimensions, such as metabolic, molecular, physiological, and phenological aspects. Appiah et al. studied four barley cultivars and their response to drought. The experiments focused on monitoring water aspects such as transpiration rate and water use efficiency and their impact on agronomic parameters such as biomass production, grain yield, and seed yield. This work allows different spring barley cultivars to be selected in response to changing water availability conditions, maximizing production.

Wheat is one of the most important crops worldwide; Yang et al. studied the effect of drought priming on winter wheat. They found that pre-exposure through drought priming stimulated the expression of genes involved in stomatal opening and closing so that treated plants could respond more quickly and efficiently at the stomatal level to the onset of drought stress.

Not only the direct study of the effect of drought on the plant is essential, but, as we have already mentioned, the development of techniques that allow us to detect the tolerance thresholds of the species as well as those that allow us to detect stress *in situ* is of vital importance. Van Laere et al. open up new ways of investigating the use of carbon 13 and its relationship to drought stress tolerance. The development of thermography and its relation to drought stress is becoming an important tool for detecting drought stress in large areas. The relationship between lack of water availability and high atmospheric evaporative demands is strongly related to stomata regulation. Stomatic movements and leaf transpiration can be visualized using digital thermography and their relationship with leaf temperature. The use of this technique can be very interesting to detect stress in large-scale crops (García-Tejero et al., 2018) or also in more specific experiments under controlled conditions in greenhouses (García-Tejero et al., 2017).

Plants lose a large amount of water through transpiration; however, another critical point in resilience to drought stress is the ability to absorb water through the roots. For this purpose, there are a large number of studies that relate drought to root development. Many recent studies have focused on the relationship between plants and mycorrhizae. Cheng et al. (2021) explain how the relationship between plants and arbuscular mycorrhizae allows for greater adaptability and tolerance of host plants to abiotic stress. This symbiotic relationship increases water absorption by the roots, as the volume of soil explored by the plants is larger. Also important in this regard are studies that analyze the relationship between plants and bacterial symbionts such as *Rhizobium* sp. Barquero et al. studied the effect of *Rhizobium leguminosarum* on the tolerance of wheat to drought stress. They showed that two of the strains studied alleviated the consequences of water stress in wheat plants by analyzing different biometric, biochemical, and gene expression parameters. This type of work is of great importance to understanding the development of mechanisms that increase the resistance of agronomic species to drought stress within the current global climate change framework.

In summary, it is necessary to achieve a balance between the sustainable use of water resources and the exploitation of large agricultural systems. The early detection of drought stress, the use of species with a high tolerance to the lack of water availability, or the use of deficit irrigation strategies are indispensable in the current context of global climate change without compromising agricultural production.

## Author contributions

AR-C: Writing – original draft, Writing – review & editing. MI: Writing – review & editing.
